# Vaccines against Genital Herpes: Where Are We?

**DOI:** 10.3390/vaccines8030420

**Published:** 2020-07-27

**Authors:** Hyeon Cheol Kim, Heung Kyu Lee

**Affiliations:** 1 Graduate School of Medical Science and Engineering, Korea Advanced Institute of Science and Technology (KAIST), Daejeon 34141, Korea; hyeoncheol@kaist.ac.kr; 2 The Center for Epidemic Preparedness, Korea Advanced Institute of Science and Technology (KAIST), Daejeon 34141, Korea

**Keywords:** genital herpes, vaccine, herpes simplex virus, HSV

## Abstract

Genital herpes is a venereal disease caused by herpes simplex virus (HSV). Although HSV symptoms can be reduced with antiviral drugs, there is no cure. Moreover, because HSV infected individuals are often unaware of their infection, it is highly likely that they will transmit HSV to their sexual partner. Once infected, an individual has to live with HSV for their entire life, and HSV infection can lead to meningitis, encephalitis, and neonatal herpes as a result of vertical transmission. In addition, HSV infection increases the rates of human immunodeficiency virus (HIV) infection and transmission. Because of the high burden of genital herpes, HSV vaccines have been developed, but none have been very successful. In this review, we discuss the current status of genital herpes vaccine development.

## 1. Introduction

Genital herpes is a common sexually transmitted disease (STD) caused by the herpes simplex virus (HSV). Because many STDs are accompanied by pain, patients seek treatment rapidly. In addition, many STDs are caused by bacteria and can be treated easily with antibiotics. However, genital herpes is often asymptomatic, thus patients have sex without knowing their infection history and are more likely to transmit the virus to their partners. Currently, there is no clear treatment for genital herpes because during a portion of its lifecycle HSV is dormant in the ganglia, and anti-HSV drugs are only effective during active virus shedding [1,2,3].

Genital herpes can be caused by both HSV-1 and HSV-2. Although the number of cases caused by HSV-1 is increasing, the main causative agent is HSV-2 [4,5]. There are 500 million people infected with HSV-2 and 140 million people infected with HSV-1, with ~23 million new cases each year [6]. Genital herpes can occur at all ages, can be painful, and may lead to multiple complications, including meningitis, encephalitis, and vertical infections from mother to fetus. Neonatal herpes arises from exposure to HSV in the vaginal mucous membranes during natural delivery. The mortality rate of neonatal herpes is 85%, and it can cause serious damage to the central nervous system (CNS) [7,8]. The main cause of genital herpes differs based on the income of the country; in high income countries (HICs), the causative agent is HSV-1, and in low-mid income countries (LMICs), the causative agent is HSV-2 [9,10]. Neonatal herpes is extremely rare in developed countries, and data on neonatal herpes in LMICs have not been evaluated in detail [11]. The incidence of neonatal herpes is expected to be significantly higher in LMICs than in HICs, due to the rapid spread of HSV-2 in LMICs [6]. Maternal HSV-2 neutralizing antibodies may prevent vertical infection to the fetus to some degree [12].

In addition, infection with HSV significantly increases the risk of human immunodeficiency virus (HIV) infection. Upon infection with HSV-2, the risk of acquiring HIV is tripled due to induction of the immune response in the genital tract, which leads to more CD4 T cells that express the CCR5 receptor, which is involved in HIV transmission and infection. Epidemiological studies have shown that in an environment where HSV-2 is spread, 25–50% of HIV infections correlate with HSV-2 infection [13,14]. The economic burden of HSV infection remains unclear, partly due to all the complications arising from HSV infection. It has been estimated that genital herpes caused by HSV costs the United States ~540 million US dollars, and this approximation only accounts for genital herpes, not the complications, thus the actual cost is likely greater [15].

Condom use during sexual intercourse minimizes HSV transmission [16], and antiviral drugs reduce viral shedding and recurrent symptoms. False-positive diagnoses can occur with the most accessible serologic test; however, the greater risk is that infected individuals are not aware of their infection status [17]. Ultimately, effective vaccine development is necessary to cope with genital herpes transmission and infection and the resulting complications. In this review, we discuss our current understanding of the immune response to HSV and the current status of vaccine development, as well as the limitations and prospects in this and related fields.

## 2. Immune Response against HSV

### 2.1. The HSV Life Cycle

**Figure 1:**
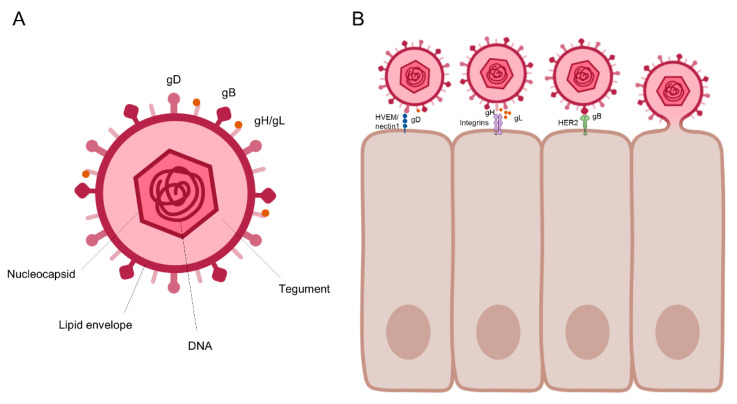
The structure of HSV and its entry (**A**) HSV virus structure. HSV has several glycoproteins in its lipid envelope, including glycoproteins, gD, gB, and gH/gL, which are known to function in cell entry. gB and gD are the most targeted molecules in the development of a vaccine against genital herpes. gB is targeted for CD4 T cell activation and gD is targeted for CD8 T cell activation. (**B**) HSV cell entry. First, gD binds to HVEM/nectin1, and this binding signals gL dissociation from gH. The gH-integrin interaction then leads to gB-HER2 binding. These serial binding interactions lead to HSV entry into the host cell. Images are created with BioRender.com.

HSV is a double-stranded DNA virus consisting of 13 glycoproteins. HSV-1 and HSV-2 are ~74% identical at the nucleotide level and structurally similar. The life cycle of HSV can be divided into lytic and latent phases. Upon transmission, HSV infects the epithelial layer of genital mucosa; HSV glycoprotein (g) D, gB, and gH/gL are involved in viral entry into epithelial cells expressing herpes virus entry mediator (HVEM) and nectin-1 (Figure 1). Upon viral entry into the cell, HSV viral DNA, including the genes necessary for replication, is transferred to the host cell nucleus [18]. There are three categories of HSV genes based on the order of expression: immediate early genes, early genes, and late genes [19]. Once the proper number of virions are formed, they leave the host cell in a manner that causes the host cell to die.

Released virions infect neighboring epithelial cells and invade the nervous system through retrograde transport along the microtubules of the axon into the neuronal cell body [20]. When HSV reaches the neuronal cell body, the viral DNA is transferred into the nucleus and maintained as an episome [19]. In the neuron, HSV only produces the latency-associated transcript (LAT) and self-replication activity is minimal, until reactivation is triggered by a specific external environmental signal, such as sunlight [21], psychological stress [22], surgical resection [23], fever and hormone [24]. When reactivated, HSV goes through anterograde transport to re-infect epithelial cells leading to cell lysis. Normally, genital herpes is more severe upon initial infection.

### 2.2. The Anti-HSV IMMUNE Response

**Figure 2:**
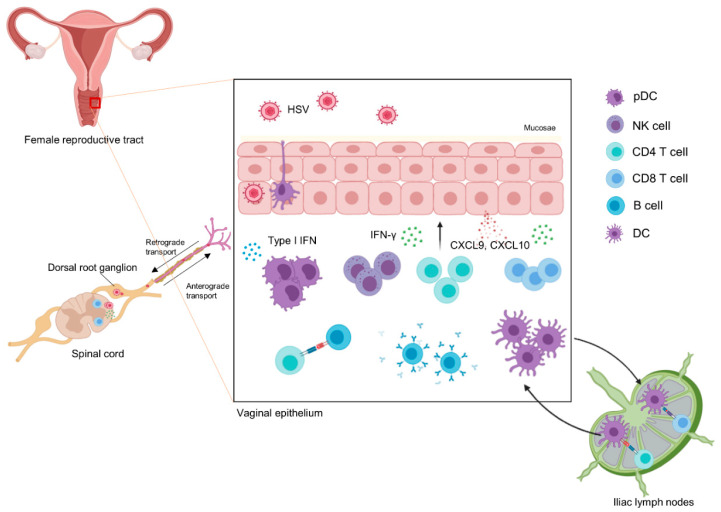
Anti-HSV immune response When HSV enters the female human body, epithelial cells recognize HSV in a PRR-dependent or PRR-independent manner. Then, pDC, NK cells, and DCs are recruited to vaginal tissue. Type I IFN, which is released from pDCs, suppresses viral replication. DCs process viral antigen, migrate to draining lymph nodes, and prime T cells. CD4 T cells appear first and induce the release of chemoattractants CXCL9 and CXCL10 via release of IFN-γ for CD8 T cell recruitment. CD4 T cells also assist B cells to generate antibody against HSV. CD8 T cells kill infected cells and control HSV reactivation. CD4 T cells orchestrate the primary response and CD8 T cells respond quickly to viral replication and reduce genital recurrence. Images are created with BioRender.com.

To develop an effective vaccine with a strong immune response, it is necessary to understand the immune response during natural infection. The initial response to HSV is induced by the innate immune system. HSV is mainly recognized by pattern-recognition receptors (PRRs), such as TLR9 for viral DNA and TLR2 for glycoproteins, and PRR-independent pathways, such as virus fusion [25,26]. In human hosts, the intrinsic defense systems include restriction factors, such as promyelocytic leukemia protein-nuclear bodies (PML-NBs) [27,28], protein inhibitor of activated STAT (PIAS) 1 [29] and 4 [30], sterile alpha motif and HD domain 1 (SAMHD1) [31], tetherin [32], and ring finger protein-8 (RNF8) [33,34]. The major role of these factors is to restrict viral proteins. However, HSV evades PML-NBs [35], PIAS1 [29], PIAS4 [30], and RNF8 [33] via E3 ubiquitin ligase ICP0, evades SAMHD1 via the conserved herpesvirus protein kinase UL13 [36], and evades tetherin via gB, gD, gH, and gL [32]. In addition, epithelial or resident immune cells recruit various immune cells, such as NK cells, plasmacytoid dendritic cells (pDCs), and dendritic cells (DCs) (Figure 2). In particular, type I interferon (IFN) released by pDCs effectively inhibits HSV replication [37,38]. In addition, genital herpes may be clinically worse and lead to complications when proper innate immunity is not induced [39,40]. Furthermore, this innate recognition of HSV is key to generating the adaptive immune response.

The kinetics of adaptive immunity show that CD4 T cells arrive first at genital tissue [41,42], primed by antigen presentation from migratory DCs [43]. The DCs that were exposed to HSV in the vagina interact with CD4 T cells in draining lymph nodes (dLNs) and iliac LNs. The CCR5-CCL5 axis is important for the inflow of CD4 T cells. Activated CD4 T cells appear within three days of infection and peak one week after infection [44]. Chemoattractants, such as CXCL9 and CXCL10, which are produced as a result of IFN-γ production by CD4 T helper type 1 (Th1) cells, recruit CD8 T cells [44]. Under homeostatic conditions, CD8 T cell infiltration into vaginal tissue is limited [44,45]. Although CD8 cytotoxic T lymphocytes (CTLs) play a role, CD4 T cells are considered more important in HSV infection because they orchestrate the anti-HSV adaptive immune response by assisting the B cell and CTL responses [46]. In mice that have been immunized, CD4 T cell depletion significantly eliminates immune memory. The effects of CTLs alone seem insignificant, but in situations where CD4 T cells are not present, the effects of CD8 T cell depletion are more significant [47].

As described above, CD4 T cells are more important during primary infection; however, CD8 T cells contribute more to immune responses in genital herpes recurrences [48,49]. Once HSV infection occurs, CD4 and CD8 T cells form clusters in vaginal tissue that remain for extended periods of time [16,50,51]. Tissue-resident memory CD8 T cells can respond to HSV very quickly, in an IFN-γ dependent manner, when the virus recurs [48,52]; this is the main immune response for recurrent infections in humans. CD8 T cells do not respond to LATs but can respond to other viral antigens [42,48,49,53,54]. Indeed, major histocompatibility complex (MHC) class I and T cell receptor (TCR) engagement was observed at the contact region between a neuron and a memory CD8 T cell [55]. Rather than being completely silent, it is thought that the virus is actually being released constantly at low levels [54]. Mathematical modeling has shown the critical role of CD8 T cells in controlling these recurrences resulting from frequent virus reactivation in a small number of infected neurons.

## 3. Status of HSV Vaccine Development

### 3.1. Overview

During HSV infection and disease, a robust T cell response is important for protection and disease control. Many vaccines have focused on inducing a strong T cell response, and currently, there are several types of vaccines. In the early days of HSV vaccine development, there was a movement to use live-attenuated HSV virus in the vaccine, but these efforts soon subsided because upon HSV vaccination, the virus stays with an individual for life. Also, at that time, there was limited information on the state of viral attenuation. In addition, early clinical trials were not randomized or double-blinded, thus vaccine effectiveness was not assessed strictly.

Two novel methods in vaccine development resolved the previous issues. One method is the elimination of some viral proteins, so viral replication is impossible or limited to one cycle [56,57,58]. The other method is to create a subunit vaccine by selecting targets that induce immune responses against HSV. In HSV vaccine history, the most famous clinical trials were conducted with subunit vaccines developed in the 1990’s. One subunit vaccine consisted of gD and gB, with the emulsion adjuvant in gB, and the other was Simplirix from GlaxoSmithKline (GSK), which consisted of gD with adjuvant system 04 (AS04). The former subunit vaccine induced a considerable amount of neutralizing antibodies but did not significantly affect recurrence [59,60]. The latter subunit vaccine showed a high efficacy of 74% in HSV-1/HSV-2 seronegative women in discordant couples but not in other serotypes or men [61]. Because this vaccine was effective in seronegative women, more clinical trials on women were performed, including the famous Herpevac trial [62] in which efficacy was only 58% against HSV-1 and 20% against HSV-2 [63]. The reason for the different trial results is unclear, but the conditions of the clinical participants differed. In the initial Simplirix trial, women in discordant couples in which sex partners were infected were the participants, but in the Herpevac trial, the participants were high-risk women who frequently visited venereal disease clinics.

Both subunit vaccines targeted gD, which is expressed on the surface of HSV and is involved in viral entry. If a strong immune response to gD is induced, it would prevent HSV from entering the host cell, and therefore, stop infection in the early stages. Because gD is known to elicit human immune responses, many vaccines candidates contained gD along with diverse adjuvants and other platforms (Table 1). Although the risk of HSV reversion is eliminated with subunit vaccines, the economic cost of manufacturing subunit vaccines remains high, so cheaper nucleic acid-based vaccine platforms were developed. These nucleic acids-based vaccines also required fewer inoculations. However, the DNA vaccine elicited weak immune responses in humans, and the RNA vaccine displayed RNA-specific instability.

### 3.2. Recent Progress

A vaccine using virus particles was made by inactivating dextran-sulfate washed HSV with formalin; monophosphoryl lipid (MPL) A/Alhydrogel was used as the adjuvant [64]. Vaccine doses were quantified by measuring the amount of protein in the vaccine, and they were subcutaneously injected into the thigh muscles of guinea pigs. This vaccine reduced the amount of virus present in the early stages of HSV infection and in lesion formation [64]. Researchers compared the efficacy of this vaccine with the recombinant gD vaccine, and there was no significant difference [64]. Nanoparticles with metal ions were also suggested as a novel vaccine platform [65]. Researchers tested zinc oxide tetrapod nanoparticles (ZOTEN), which reduce HSV vaginal infection, as a vaccine platform for genital herpes [66]. Mice given ZOTEN and HSV intravaginally showed milder pathological symptoms, but a similar anti-HSV immune response, than mice given HSV virus only [65] indicating that ZOTEN has potential as a prophylactic HSV vaccine.

The clinical failure of the subunit vaccines suggested the possibility that a potent antigen was lacking in the vaccines. Although several antigen screening processes were performed [67,68,69,70,71], they may have missed antigens that induce a robust immune response. Therefore, a live-attenuated vaccine containing HSV with a gK deletion, which prevents entry into neurons, was developed [72]. The gK-deleted virus cannot invade the nervous system, but it also requires a special complementing cell line, VK302, for replication [72]. Thus, the VC2 vaccine, which contains HSV with a partial gK deletion and a UL20 deletion, was created [73]. The VC2 vaccine reduced acute and recurrent HSV-2 disease, viral shedding, and the amount of virus detected in neurons [74]. However, the VC2 vaccine is specific for neural infection, so it is more suitable as a preventive vaccine [74].

There are also many nucleic acid-based vaccines in which gD and other antigens are targeted. Because gD mainly produces CD4 T cell-related responses, proteins UL25 and gB, which contain the CD8 T cell specific epitopes, were selected as additional targets to boost the CTL response [47,75]. In addition, molecules expressed in the early lytic phase, such as ICP0, ICP4, and UL39, were targeted to raise the overall T cell response [47]. Because of the low immunogenicity of DNA vaccines, cytokines were also used. A DNA vaccine containing interleukin 28B (IL-28B), which is known to enhance cellular and humoral responses in mouse models, showed a preventive effect against genital herpes in a guinea pig model [75]. A DNA vaccine containing IL-12, IL-21, and macrophage inflammatory protein-1 alpha (MIP-1α) induced a virus-specific T cell response in vaginal tissue and eliminated the virus fairly well, indicating that it has a preventive effect against primary infection in a mouse model [47].

Another vaccine targeting similar antigens used the recombinant adenovirus type 5 (Ad5) vector platform [76]. Ad5 is known to induce a robust antibody reaction and T cell response to inserted genes. By inserting gD and UL25 genes into Ad5, induction of appropriate CD4 T cell and CD8 T cell responses were expected [42,53,67,77]. This vaccine was injected into the muscles of mice and effectively prevented genital herpes by inducing IFN-γ production by T cells [76].

In addition to a change in the platform, there is interest in exploring new HSV vaccine targets. A vaccine targeting the HSV-2 specific protein gG2 may solve the problem of low vaccine efficacy in HSV-1 seropositive persons. The protein is divided into the major part (mgG2) and the small secreted part (sgG2) during viral processing [78,79,80], and recent studies have shown that sgG2 aids in chemotaxis and related immune cell signaling [81,82]. The gG2 vaccine induced an especially robust CD4 T cell response and prevented genital HSV infection. Due to the initial strong response, the amount of virus detected in the nervous system was low [83].

A subunit vaccine was also used to target multiple glycoproteins at once. The existing vaccine for gD was limited due to the immune evasion mechanism of HSV, which involves HSV gC and gE; therefore, gC and gE were added to the vaccine to suppress this evasion. Complement protein C3, which is the most abundant protein in the complement cascade, is cleaved to C3b, and C3b helps neutralize HSV. HSV gC binding to C3b hampers this neutralization [84]. HSV gE binds to the Fc region of IgG antibodies blocking their action [85]. The gD2/gC2/gE2 trivalent subunit vaccine showed immunogenicity in rhesus macaques and preventive and therapeutic effects in guinea pigs [86,87], as well as protection against genital herpes caused by HSV-1 in a guinea pig model [88]. To overcome the shortcomings of the subunit vaccine, an RNA platform was used [89]. These trivalent subunit and RNA vaccine displayed preventive effects in mouse and guinea pig models and induced considerable antibody and T cell responses [86,88,89,90].

## 4. Limitations & Future Directions

For approximately a century, various efforts have been made to develop a genital herpes vaccine but some limitations remain. First, the mouse is a convenient experimental animal, but the pattern of human HSV disease progression is not reproduced in the mouse [91,92]. HSV lesions cause mice to die, but HSV is not lethal in humans. Thus, a genital herpes vaccine that is efficacious in a mouse animal model cannot be translated to humans.

For this reason, researchers use the mouse model for prophylactic vaccines and the guinea pig model for therapeutic vaccines. The guinea pigs are an outbred strain that represents the human population well, but there are still limitations for the study of vaccine mechanisms [93,94]. Because experimental materials and methods for guinea pigs are limited, it is difficult to conduct experiments with gene knockouts to evaluate mechanisms [95,96]. Inbred guinea pigs exist, but their availability is low. In addition to mice and guinea pigs, there are other experimental animal models for genital herpes studies. Cotton rats are easily infected intravaginally with HSV-2, they do not need to be treated with medroxy progesterone acetate (MPA), and recurrence can happen spontaneously [97]. However, like the guinea pig model, there are few experimental materials and methods to investigate the immune response in the cotton rat model. To narrow the gap between pre-clinical studies and clinical trials, rhesus macaques and owl monkeys could be used. In rhesus macaques, only 10% of infected animals show genital lesions [98], but the infection is fatal in owl monkeys [99]. Although they are highly similar to humans, the rhesus macaque and owl monkey models are too costly. A suitable experimental animal model still needs to be established to more reliably determine the efficacy of a genital herpes vaccine.

The gender of the experimental animal also needs to be considered. It is more difficult to infect a male with a protruding reproductive structure than a female with inward genital organs. Thus, most experiments are conducted on female animals, which explains why most of the HSV vaccines work well in women but poorly in men. For the development of a proper genital herpes vaccine, an appropriate male animal model should be established. Nevertheless, gender-specific differences cannot be ignored and must be noted. The presence of different sex hormones and the different genital structures may affect vaccine effectiveness. Genital structural differences lead to differences in the experimental infection process and to environmental differences of the infected areas. In the vagina, relatively thick peripheral tissue may allow normal residence of a significant number of immune cells. On the other hand, there is little extra space on a penis for the residence of immune cells. In addition, there are differences between penile and vaginal microbiota. Because male genitalia are exposed to air, penile skin microbiota are likely aerobic microorganisms. Because of its inward structure, the vaginal microbiota is exposed to less oxygen and may be suitable for anaerobic microorganisms. These environmental factors may alter the effectiveness of vaccines according to gender.

Researchers have attempted to develop prophylactic and therapeutic vaccines against genital herpes using several vaccine platforms and adjuvants. The adjuvants are primarily used to induce more robust T cell and antibody responses against HSV. As in other vaccines, alum, MF59, MPL, AS04, and QS21 have been used as adjuvants in HSV vaccines (Table 1). Most of these adjuvants stimulate innate immune cells or dendritic cells to induce a potent and long-lasting immune response. Thus, for a successful vaccine, the proper adjuvant that supports and magnifies the efficacy of the vaccine should be used. It should also be noted that there are some differences between mice and human immune cells. Murine XCR1+ DCs express TLR3, 4, and 9 [100], whereas the human counterparts only express TLR3 [101]. Thus, a TLR4 agonist, such as MPL or glucopyranosyl lipid A (GLA), would not target human XCR1+ DCs directly. Adjuvants also affect the induction of immune cells, for example MPL induces Th1 cells and QS21 induces memory CD8 T cells [102]. Thus, the mode of action and the nature of the target immune system are important factors to consider when selecting the proper adjuvant.

The Simplirix HSV-2 subunit vaccine is only efficacious for HSV-1 and HSV-2 seronegative women. Based on the similarities between HSV-1 and HSV-2, one vaccine should have an effect on both viruses. However, because a considerable number of people have already been exposed to HSV-1, a vaccine that targets a specific protein in HSV-2 would be beneficial. The viral protein gD is a good target because it is involved in host intracellular access. In addition, it is expressed in both HSV-1 and HSV-2 and can elicit strong responses from subjects who have never been exposed to either virus. However, if a person has previously been infected with one of the viruses, a strong immune response will not be induced. Thus, a more specific target should be selected [83]. 

## 5. Conclusions

When considering targets, proteins involved in viral entry are candidates for preventive vaccines; however, genital herpes preventative vaccine studies have not been completely successful. Thus, novel vaccines that target early generated molecules or immune evasion molecules are being developed [85,86,87]. Despite a great deal of research, we still do not have a highly effective vaccine to counter genital herpes. With continued research, we look forward to the day when HSV and genital herpes will be preventable.

## Figures and Tables

**Table 1:** Genital herpes vaccine clinical trial status table.

Classification	Candidate	Company	Composition	Status	Phase	Identifier ^1^	Completion
Subunit vaccine	gD2	Novartis (previous Chiron)	gD2 plus alum	Stopped after Phase II trial	Phase I	-	1992
	gD2/gB2		gD2 and gB2 plus MF59		Phase II	-	1997
	Simplirix/Herpevac	Glaxo-SmithKline (GSK)	gD2 and AS04 (dMPL)	Stopped after Phase III trial	Phase III	NCT00057330	2009
	GEN-003	Genocea	gD2 and Matrix M2	Stopped after Phase II trial	Phase I/II	NCT01667341	2014
					Phase II	NCT02114060	2016
						NCT02300142	2016
						NCT02515175	2017
						NCT03146403	2018
	HerpV (previous AG-707)	Agenus	Peptide vaccine + QS-21 Stimulon	Stopped after Phase II trial	Phase I	NCT00231049	2006
					Phase II	NCT01687595	2015
Live-attenuated vaccine	ICP10ΔPK	AuRx	ICP10ΔPK	Stopped after Phase I/IIa trial	Phase I/IIa	-	2002
	HSV529	Sanofi Pasteur	Replication defective HSV2, UL5, UL29 deletion	Phase II trial ongoing	Phase I	NCT01915212	2017
						NCT02571166	2018
					Phase I/II	NCT04222985	2023
DNA vaccine	pPJV7630	Powder-Med	Ubiquitinated and unmodified gD2	Stopped after Phase I trial	Phase I	NCT00274300	2005
				-	Phase I	NCT00310271	2006
	VCL-HB01	Vical	gD2 +/− UL46 and Vaxfectin DNA vaccine	Stopped after Phase II trial	Phase I/II	NCT02030301	2016
				-	Phase II	NCT02837575	2018
	COR-1	Anteris (previous ADMEDUS)	gD2 codon optimized DNA vaccine	Stopped after Phase I/IIa trial	Phase I/IIa	-	2017

^1^ This identifier means the identifier used at https://clinicaltrials.gov/.

## References

[1] Gupta R., Wald A., Krantz E., Selke S., Warren T., Vargas-Cortes M., Miller G., Corey L. (2004). Valacyclovir and acyclovir for suppression of shedding of herpes simplex virus in the genital tract. J. Infect. Dis..

[2] Corey L., Wald A., Patel R., Sacks S.L., Tyring S.K., Warren T., Douglas J.M., Paavonen J., Morrow R.A., Beutner K.R. (2004). Once-daily valacyclovir to reduce the risk of transmission of genital herpes. N. Engl. J. Med..

[3] Martens M.G., Fife K.H., Leone P.A., Dix L.P., Brennan C.A. (2009). Once daily valacyclovir for reducing viral shedding in subjects newly diagnosed with genital herpes. Infect. Dis. Obstet. Gynecol..

[4] Hofstetter A.M., Rosenthal S.L., Stanberry L.R. (2014). Current thinking on genital herpes. Curr. Opin. Infect. Dis..

[5] Bernstein D.I., Bellamy A.R., Hook E.W., Levin M.J., Wald A., Ewell M.G., Wolff P.A., Deal C.D., Heineman T.C., Dubin G. (2013). Epidemiology, clinical presentation, and antibody response to primary infection with herpes simplex virus type 1 and type 2 in young women. Clin. Infect. Dis..

[6] Looker K.J., Garnett G.P., Schmid G.P. (2008). An estimate of the global prevalence and incidence of herpes simplex virus type 2 infection. Bull. World Health Organ..

[7] Whitley R.J., Nahmias A.J., Soong S.J., Galasso G.G., Fleming C.L., Alford C.A. (1980). Vidarabine therapy of neonatal herpes simplex virus infection. Pediatrics.

[8] Kimberlin D.W. (2004). Neonatal herpes simplex infection. Clin. Microbiol. Rev..

[9] Morris S.R., Bauer H.M., Samuel M.C., Gallagher D., Bolan G. (2008). Neonatal herpes morbidity and mortality in California, 1995–2003. Sex. Transm. Dis..

[10] Ryder N., Jin F., McNulty A.M., Grulich A.E., Donovan B. (2009). Increasing role of herpes simplex virus type 1 in first-episode anogenital herpes in heterosexual women and younger men who have sex with men, 1992–2006. Sex. Transm. Infect..

[11] Flagg E.W., Weinstock H. (2011). Incidence of neonatal herpes simplex virus infections in the United States, 2006. Pediatrics.

[12] Prober C.G., Sullender W.M., Yasukawa L.L., Au D.S., Yeager A.S., Arvin A.M. (1987). Low risk of herpes simplex virus infections in neonates exposed to the virus at the time of vaginal delivery to mothers with recurrent genital herpes simplex virus infections. N. Engl. J. Med..

[13] Freeman E.E., Orroth K.K., White R.G., Glynn J.R., Bakker R., Boily M.C., Habbema D., Buve A., Hayes R. (2007). Proportion of new HIV infections attributable to herpes simplex 2 increases over time: Simulations of the changing role of sexually transmitted infections in sub-Saharan African HIV epidemics. Sex. Transm. Infect..

[14] Masese L., Baeten J.M., Richardson B.A., Bukusi E., John-Stewart G., Graham S.M., Shafi J., Kiarie J., Overbaugh J., McClelland R.S. (2015). Changes in the contribution of genital tract infections to HIV acquisition among Kenyan high-risk women from 1993 to 2012. AIDS.

[15] Owusu-Edusei K., Chesson H.W., Gift T.L., Tao G., Mahajan R., Ocfemia M.C., Kent C.K. (2013). The estimated direct medical cost of selected sexually transmitted infections in the United States, 2008. Sex. Transm. Dis..

[16] Martin E.T., Krantz E., Gottlieb S.L., Magaret A.S., Langenberg A., Stanberry L., Kamb M., Wald A. (2009). A pooled analysis of the effect of condoms in preventing HSV-2 acquisition. Arch. Intern. Med..

[17] Lingappa J., Nakku-Joloba E., Magaret A., Friedrich D., Dragavon J., Kambugu F., Joloba M., Whalen C., Coombs R., Celum C. (2010). Sensitivity and specificity of herpes simplex virus-2 serological assays among HIV-infected and uninfected urban Ugandans. Int. J. STD AIDS.

[18] Campadelli-Fiume G., Menotti L., Avitabile E., Gianni T. (2012). Viral and cellular contributions to herpes simplex virus entry into the cell. Curr. Opin. Virol..

[19] Knipe D.M., Cliffe A. (2008). Chromatin control of herpes simplex virus lytic and latent infection. Nat. Rev. Microbiol..

[20] Smith G. (2012). Herpesvirus transport to the nervous system and back again. Annu. Rev. Microbiol..

[21] Spruance S.L. (1988). Cutaneous herpes simplex virus lesions induced by ultraviolet radiation. A review of model systems and prophylactic therapy with oral acyclovir. Am. J. Med..

[22] Chida Y., Mao X. (2009). Does psychosocial stress predict symptomatic herpes simplex virus recurrence? A meta-analytic investigation on prospective studies. Brain Behav. Immun..

[23] El Hayderi L., Delvenne P., Rompen E., Senterre J.M., Nikkels A.F. (2013). Herpes simplex virus reactivation and dental procedures. Clin. Oral Investig..

[24] Wilson A.C., Mohr I. (2012). A cultured affair: HSV latency and reactivation in neurons. Trends Microbiol..

[25] Kinnebrew M.A., Buffie C.G., Diehl G.E., Zenewicz L.A., Leiner I., Hohl T.M., Flavell R.A., Littman D.R., Pamer E.G. (2012). Interleukin 23 production by intestinal CD103(+)CD11b(+) dendritic cells in response to bacterial flagellin enhances mucosal innate immune defense. Immunity.

[26] Medzhitov R., Janeway C. (2000). Innate immune recognition: Mechanisms and pathways. Immunol. Rev..

[27] Everett R.D., Rechter S., Papior P., Tavalai N., Stamminger T., Orr A. (2006). PML contributes to a cellular mechanism of repression of herpes simplex virus type 1 infection that is inactivated by ICP0. J. Virol..

[28] Everett R.D., Parada C., Gripon P., Sirma H., Orr A. (2008). Replication of ICP0-null mutant herpes simplex virus type 1 is restricted by both PML and Sp100. J. Virol..

[29] Brown J.R., Conn K.L., Wasson P., Charman M., Tong L., Grant K., McFarlane S., Boutell C. (2016). SUMO Ligase Protein Inhibitor of Activated STAT1 (PIAS1) Is a Constituent Promyelocytic Leukemia Nuclear Body Protein That Contributes to the Intrinsic Antiviral Immune Response to Herpes Simplex Virus 1. J. Virol..

[30] Conn K.L., Wasson P., McFarlane S., Tong L., Brown J.R., Grant K.G., Domingues P., Boutell C. (2016). Novel Role for Protein Inhibitor of Activated STAT 4 (PIAS4) in the Restriction of Herpes Simplex Virus 1 by the Cellular Intrinsic Antiviral Immune Response. J. Virol..

[31] Kim E.T., White T.E., Brandariz-Nunez A., Diaz-Griffero F., Weitzman M.D. (2013). SAMHD1 restricts herpes simplex virus 1 in macrophages by limiting DNA replication. J. Virol..

[32] Liu Y., Luo S., He S., Zhang M., Wang P., Li C., Huang W., Hu B., Griffin G.E., Shattock R.J. (2015). Tetherin restricts HSV-2 release and is counteracted by multiple viral glycoproteins. Virology.

[33] Lilley C.E., Chaurushiya M.S., Boutell C., Landry S., Suh J., Panier S., Everett R.D., Stewart G.S., Durocher D., Weitzman M.D. (2010). A viral E3 ligase targets RNF8 and RNF168 to control histone ubiquitination and DNA damage responses. EMBO J..

[34] Lilley C.E., Chaurushiya M.S., Boutell C., Everett R.D., Weitzman M.D. (2011). The intrinsic antiviral defense to incoming HSV-1 genomes includes specific DNA repair proteins and is counteracted by the viral protein ICP0. PLoS Pathog..

[35] Maul G.G., Everett R.D. (1994). The nuclear location of PML, a cellular member of the C3HC4 zinc-binding domain protein family, is rearranged during herpes simplex virus infection by the C3HC4 viral protein ICP0. J. Gen. Virol..

[36] Zhang K., Lv D.W., Li R. (2019). Conserved Herpesvirus Protein Kinases Target SAMHD1 to Facilitate Virus Replication. Cell Rep..

[37] Sainz B., Halford W.P. (2002). Alpha/Beta interferon and gamma interferon synergize to inhibit the replication of herpes simplex virus type 1. J. Virol..

[38] Leventon-Kriss S., Movshovitz M., Smetana Z., Shewach-Millet M., Doerner T., Gotlieb-Stematsky T. (1987). Sensitivity in vitro of herpes simplex virus isolates to human fibroblast interferon. Med. Microbiol. Immunol..

[39] Sancho-Shimizu V., Perez de Diego R., Lorenzo L., Halwani R., Alangari A., Israelsson E., Fabrega S., Cardon A., Maluenda J., Tatematsu M. (2011). Herpes simplex encephalitis in children with autosomal recessive and dominant TRIF deficiency. J. Clin. Investig..

[40] Dropulic L.K., Cohen J.I. (2011). Severe viral infections and primary immunodeficiencies. Clin. Infect. Dis..

[41] Cunningham A.L., Turner R.R., Miller A.C., Para M.F., Merigan T.C. (1985). Evolution of recurrent herpes simplex lesions. An immunohistologic study. J. Clin. Investig..

[42] Koelle D.M., Posavad C.M., Barnum G.R., Johnson M.L., Frank J.M., Corey L. (1998). Clearance of HSV-2 from recurrent genital lesions correlates with infiltration of HSV-specific cytotoxic T lymphocytes. J. Clin. Investig..

[43] Iwasaki A. (2007). Mucosal dendritic cells. Annu. Rev. Immunol..

[44] Nakanishi Y., Lu B., Gerard C., Iwasaki A. (2009). CD8(+) T lymphocyte mobilization to virus-infected tissue requires CD4(+) T-cell help. Nature.

[45] Wong S.B., Bos R., Sherman L.A. (2008). Tumor-specific CD4+ T cells render the tumor environment permissive for infiltration by low-avidity CD8+ T cells. J. Immunol..

[46] Swain S.L., McKinstry K.K., Strutt T.M. (2012). Expanding roles for CD4(+) T cells in immunity to viruses. Nat. Rev. Immunol..

[47] Kim H.C., Oh D.S., Park J.H., Kim H.J., Seo Y.B., Yoo H.J., Jang H.S., Shin J., Kim C.W., Kwon M.S. (2020). Multivalent DNA vaccine protects against genital herpes by T-cell immune induction in vaginal mucosa. Antivir. Res..

[48] Hoshino Y., Pesnicak L., Cohen J.I., Straus S.E. (2007). Rates of reactivation of latent herpes simplex virus from mouse trigeminal ganglia ex vivo correlate directly with viral load and inversely with number of infiltrating CD8+ T cells. J. Virol..

[49] Simmons A., Tscharke D.C. (1992). Anti-CD8 impairs clearance of herpes simplex virus from the nervous system: Implications for the fate of virally infected neurons. J. Exp. Med..

[50] Iijima N., Linehan M.M., Zamora M., Butkus D., Dunn R., Kehry M.R., Laufer T.M., Iwasaki A. (2008). Dendritic cells and B cells maximize mucosal Th1 memory response to herpes simplex virus. J. Exp. Med..

[51] Sajic D., Patrick A.J., Rosenthal K.L. (2005). Mucosal delivery of CpG oligodeoxynucleotides expands functional dendritic cells and macrophages in the vagina. Immunology.

[52] Schiffer J.T., Abu-Raddad L., Mark K.E., Zhu J., Selke S., Koelle D.M., Wald A., Corey L. (2010). Mucosal host immune response predicts the severity and duration of herpes simplex virus-2 genital tract shedding episodes. Proc. Natl. Acad. Sci. USA.

[53] Zhu J., Koelle D.M., Cao J., Vazquez J., Huang M.L., Hladik F., Wald A., Corey L. (2007). Virus-specific CD8+ T cells accumulate near sensory nerve endings in genital skin during subclinical HSV-2 reactivation. J. Exp. Med..

[54] Schiffer J.T., Abu-Raddad L., Mark K.E., Zhu J., Selke S., Magaret A., Wald A., Corey L. (2009). Frequent release of low amounts of herpes simplex virus from neurons: Results of a mathematical model. Sci. Transl. Med..

[55] Khanna K.M., Bonneau R.H., Kinchington P.R., Hendricks R.L. (2003). Herpes simplex virus-specific memory CD8+ T cells are selectively activated and retained in latently infected sensory ganglia. Immunity.

[56] Skinner G.R., Turyk M.E., Benson C.A., Wilbanks G.D., Heseltine P., Galpin J., Kaufman R., Goldberg L., Hartley C.E., Buchan A. (1997). The efficacy and safety of Skinner herpes simplex vaccine towards modulation of herpes genitalis; report of a prospective double-blind placebo-controlled trial. Med. Microbiol. Immunol..

[57] Aurelian L., Kokuba H., Smith C.C. (1999). Vaccine potential of a herpes simplex virus type 2 mutant deleted in the PK domain of the large subunit of ribonucleotide reductase (ICP10). Vaccine.

[58] Casanova G., Cancela R., Alonzo L., Benuto R., Magana Mdel C., Hurley D.R., Fishbein E., Lara C., Gonzalez T., Ponce R. (2002). A double-blind study of the efficacy and safety of the ICP10deltaPK vaccine against recurrent genital HSV-2 infections. Cutis.

[59] Straus S.E., Wald A., Kost R.G., McKenzie R., Langenberg A.G., Hohman P., Lekstrom J., Cox E., Nakamura M., Sekulovich R. (1997). Immunotherapy of recurrent genital herpes with recombinant herpes simplex virus type 2 glycoproteins D and B: Results of a placebo-controlled vaccine trial. J. Infect. Dis..

[60] Corey L., Langenberg A.G., Ashley R., Sekulovich R.E., Izu A.E., Douglas J.M., Handsfield H.H., Warren T., Marr L., Tyring S. (1999). Recombinant glycoprotein vaccine for the prevention of genital HSV-2 infection: Two randomized controlled trials. Chiron HSV Vaccine Study Group. JAMA.

[61] Stanberry L.R., Spruance S.L., Cunningham A.L., Bernstein D.I., Mindel A., Sacks S., Tyring S., Aoki F.Y., Slaoui M., Denis M. (2002). Glycoprotein-D-adjuvant vaccine to prevent genital herpes. N. Engl. J. Med..

[62] Belshe R.B., Leone P.A., Bernstein D.I., Wald A., Levin M.J., Stapleton J.T., Gorfinkel I., Morrow R.L., Ewell M.G., Stokes-Riner A. (2012). Efficacy results of a trial of a herpes simplex vaccine. N. Engl. J. Med..

[63] Cohen J. (2010). Immunology. Painful failure of promising genital herpes vaccine. Science.

[64] Bernstein D.I., Morello C.S., Cardin R.D., Bravo F.J., Kraynyak K.A., Spector D.H. (2020). A vaccine containing highly purified virus particles in adjuvant provides high level protection against genital infection and disease in guinea pigs challenged intravaginally with homologous and heterologous strains of herpes simplex virus type 2. Vaccine.

[65] Agelidis A., Koujah L., Suryawanshi R., Yadavalli T., Mishra Y.K., Adelung R., Shukla D. (2019). An Intra-Vaginal Zinc Oxide Tetrapod Nanoparticles (ZOTEN) and Genital Herpesvirus Cocktail Can Provide a Novel Platform for Live Virus Vaccine. Front. Immunol..

[66] Antoine T.E., Mishra Y.K., Trigilio J., Tiwari V., Adelung R., Shukla D. (2012). Prophylactic, therapeutic and neutralizing effects of zinc oxide tetrapod structures against herpes simplex virus type-2 infection. Antivir. Res..

[67] Hosken N., McGowan P., Meier A., Koelle D.M., Sleath P., Wagener F., Elliott M., Grabstein K., Posavad C., Corey L. (2006). Diversity of the CD8 + T-cell response to herpes simplex virus type 2 proteins among persons with genital herpes. J. Virol..

[68] Laing K.J., Magaret A.S., Mueller D.E., Zhao L., Johnston C., De Rosa S.C., Koelle D.M., Wald A., Corey L. (2010). Diversity in CD8 (+) T cell function and epitope breadth among persons with genital herpes. J. Clin. Immunol..

[69] Koelle D.M., Liu Z., McClurkan C.L., Cevallos R.C., Vieira J., Hosken N.A., Meseda C.A., Snow D.C., Wald A., Corey L. (2003). Immunodominance among herpes simplex virus-specific CD8 T cells expressing a tissue-specific homing receptor. Proc. Natl. Acad. Sci. USA.

[70] Koelle D.M., Chen H.B., Gavin M.A., Wald A., Kwok W.W., Corey L. (2001). CD8 CTL from genital herpes simplex lesions: Recognition of viral tegument and immediate early proteins and lysis of infected cutaneous cells. J. Immunol..

[71] Koelle D.M., Frank J.M., Johnson M.L., Kwok W.W. (1998). Recognition of herpes simplex virus type 2 tegument proteins by CD4 T cells infiltrating human genital herpes lesions. J. Virol..

[72] Iyer A.V., Pahar B., Chouljenko V.N., Walker J.D., Stanfield B., Kousoulas K.G. (2013). Single dose of glycoprotein K (gK)-deleted HSV-1 live-attenuated virus protects mice against lethal vaginal challenge with HSV-1 and HSV-2 and induces lasting T cell memory immune responses. Virol. J..

[73] Stanfield B.A., Rider P.J.F., Caskey J., Del Piero F., Kousoulas K.G. (2018). Intramuscular vaccination of guinea pigs with the live-attenuated human herpes simplex vaccine VC2 stimulates a transcriptional profile of vaginal Th17 and regulatory Tr1 responses. Vaccine.

[74] Bernstein D.I., Pullum D.A., Cardin R.D., Bravo F.J., Dixon D.A., Kousoulas K.G. (2019). The HSV-1 live attenuated VC2 vaccine provides protection against HSV-2 genital infection in the guinea pig model of genital herpes. Vaccine.

[75] Zhou Y., Wang Z., Xu Y., Zhang Z., Hua R., Liu W., Jiang C., Chen Y., Yang W., Kong W. (2017). Optimized DNA Vaccine Enhanced by Adjuvant IL28B Induces Protective Immune Responses Against Herpes Simplex Virus Type 2 in Mice. Viral Immunol..

[76] Liu W., Zhou Y., Wang Z., Zhang Z., Wang Q., Su W., Chen Y., Zhang Y., Gao F., Jiang C. (2017). Evaluation of recombinant adenovirus vaccines based on glycoprotein D and truncated UL25 against herpes simplex virus type 2 in mice. Microbiol. Immunol..

[77] Ogasawara M., Suzutani T., Yoshida I., Azuma M. (2001). Role of the UL25 gene product in packaging DNA into the herpes simplex virus capsid: Location of UL25 product in the capsid and demonstration that it binds DNA. J. Virol..

[78] Su H.K., Eberle R., Courtney R.J. (1987). Processing of the herpes simplex virus type 2 glycoprotein gG-2 results in secretion of a 34,000-Mr cleavage product. J. Virol..

[79] Balachandran N., Hutt-Fletcher L.M. (1985). Synthesis and processing of glycoprotein gG of herpes simplex virus type 2. J. Virol..

[80] Olofsson S., Lundstrom M., Marsden H., Jeansson S., Vahlne A. (1986). Characterization of a herpes simplex virus type 2-specified glycoprotein with affinity for N-acetylgalactosamine-specific lectins and its identification as g92K or gG. J. Gen. Virol..

[81] Viejo-Borbolla A., Martinez-Martin N., Nel H.J., Rueda P., Martin R., Blanco S., Arenzana-Seisdedos F., Thelen M., Fallon P.G., Alcami A. (2012). Enhancement of chemokine function as an immunomodulatory strategy employed by human herpesviruses. PLoS Pathog..

[82] Martinez-Martin N., Viejo-Borbolla A., Martin R., Blanco S., Benovic J.L., Thelen M., Alcami A. (2015). Herpes simplex virus enhances chemokine function through modulation of receptor trafficking and oligomerization. Nat. Commun..

[83] Onnheim K., Ekblad M., Gorander S., Bergstrom T., Liljeqvist J.A. (2016). Vaccination with the Secreted Glycoprotein G of Herpes Simplex Virus 2 Induces Protective Immunity after Genital Infection. Viruses.

[84] Awasthi S., Lubinski J.M., Shaw C.E., Barrett S.M., Cai M., Wang F., Betts M., Kingsley S., Distefano D.J., Balliet J.W. (2011). Immunization with a vaccine combining herpes simplex virus 2 (HSV-2) glycoprotein C (gC) and gD subunits improves the protection of dorsal root ganglia in mice and reduces the frequency of recurrent vaginal shedding of HSV-2 DNA in guinea pigs compared to immunization with gD alone. J. Virol..

[85] Awasthi S., Huang J., Shaw C., Friedman H.M. (2014). Blocking herpes simplex virus 2 glycoprotein E immune evasion as an approach to enhance efficacy of a trivalent subunit antigen vaccine for genital herpes. J. Virol..

[86] Awasthi S., Hook L.M., Shaw C.E., Pahar B., Stagray J.A., Liu D., Veazey R.S., Friedman H.M. (2017). An HSV-2 Trivalent Vaccine Is Immunogenic in Rhesus Macaques and Highly Efficacious in Guinea Pigs. PLoS Pathog..

[87] Awasthi S., Hook L.M., Shaw C.E., Friedman H.M. (2017). A trivalent subunit antigen glycoprotein vaccine as immunotherapy for genital herpes in the guinea pig genital infection model. Hum. Vaccin Immunother..

[88] Egan K., Hook L.M., Naughton A., Friedman H.M., Awasthi S. (2020). Herpes simplex virus type 2 trivalent protein vaccine containing glycoproteins C, D and E protects guinea pigs against HSV-1 genital infection. Hum. Vaccin Immunother..

[89] Hook L.M., Awasthi S., Dubin J., Flechtner J., Long D., Friedman H.M. (2019). A trivalent gC2/gD2/gE2 vaccine for herpes simplex virus generates antibody responses that block immune evasion domains on gC2 better than natural infection. Vaccine.

[90] Awasthi S., Hook L.M., Pardi N., Wang F., Myles A., Cancro M.P., Cohen G.H., Weissman D., Friedman H.M. (2019). Nucleoside-modified mRNA encoding HSV-2 glycoproteins C, D, and E prevents clinical and subclinical genital herpes. Sci. Immunol..

[91] Kollias C.M., Huneke R.B., Wigdahl B., Jennings S.R. (2015). Animal models of herpes simplex virus immunity and pathogenesis. J. Neurovirol..

[92] Sawtell N.M., Thompson R.L. (1992). Herpes simplex virus type 1 latency-associated transcription unit promotes anatomical site-dependent establishment and reactivation from latency. J. Virol..

[93] Hensel M.T., Marshall J.D., Dorwart M.R., Heeke D.S., Rao E., Tummala P., Yu L., Cohen G.H., Eisenberg R.J., Sloan D.D. (2017). Prophylactic Herpes Simplex Virus 2 (HSV-2) Vaccines Adjuvanted with Stable Emulsion and Toll-Like Receptor 9 Agonist Induce a Robust HSV-2-Specific Cell-Mediated Immune Response, Protect against Symptomatic Disease, and Reduce the Latent Viral Reservoir. J. Virol..

[94] Bernstein D.I., Cardin R.D., Bravo F.J., Awasthi S., Lu P., Pullum D.A., Dixon D.A., Iwasaki A., Friedman H.M. (2019). Successful application of prime and pull strategy for a therapeutic HSV vaccine. NPJ Vaccines.

[95] Stanberry L.R., Kern E.R., Richards J.T., Abbott T.M., Overall J.C. (1982). Genital herpes in guinea pigs: Pathogenesis of the primary infection and description of recurrent disease. J. Infect. Dis..

[96] Stanberry L.R., Kern E.R., Richards J.T., Overall J.C. (1985). Recurrent genital herpes simplex virus infection in guinea pigs. Intervirology.

[97] Yim K.C., Carroll C.J., Tuyama A., Cheshenko N., Carlucci M.J., Porter D.D., Prince G.A., Herold B.C. (2005). The cotton rat provides a novel model to study genital herpes infection and to evaluate preventive strategies. J. Virol..

[98] Crostarosa F., Aravantinou M., Akpogheneta O.J., Jasny E., Shaw A., Kenney J., Piatak M., Lifson J.D., Teitelbaum A., Hu L. (2009). A macaque model to study vaginal HSV-2/immunodeficiency virus co-infection and the impact of HSV-2 on microbicide efficacy. PLoS ONE.

[99] Melendez L.V., Espana C., Hunt R.D., Daniel M.D., Garcia F.G. (1969). Natural herpes simplex infection in the owl monkey (Aotus trivirgatus). Lab. Anim. Care.

[100] Edwards A.D., Diebold S.S., Slack E.M., Tomizawa H., Hemmi H., Kaisho T., Akira S., Reis e Sousa C. (2003). Toll-like receptor expression in murine DC subsets: Lack of TLR7 expression by CD8 alpha+ DC correlates with unresponsiveness to imidazoquinolines. Eur. J. Immunol..

[101] Jongbloed S.L., Kassianos A.J., McDonald K.J., Clark G.J., Ju X., Angel C.E., Chen C.J., Dunbar P.R., Wadley R.B., Jeet V. (2010). Human CD141+ (BDCA-3)+ dendritic cells (DCs) represent a unique myeloid DC subset that cross-presents necrotic cell antigens. J. Exp. Med..

[102] Wald A., Koelle D.M., Fife K., Warren T., Leclair K., Chicz R.M., Monks S., Levey D.L., Musselli C., Srivastava P.K. (2011). Safety and immunogenicity of long HSV-2 peptides complexed with rhHsc70 in HSV-2 seropositive persons. Vaccine.

